# Phytogenic feed additives alleviate pathogenic *Escherichia coli*-induced intestinal damage through improving barrier integrity and inhibiting inflammation in weaned pigs

**DOI:** 10.1186/s40104-022-00750-y

**Published:** 2022-09-02

**Authors:** Se Yeon Chang, Min Ho Song, Ji Hwan Lee, Han Jin Oh, Yong Ju Kim, Jae Woo An, Young Bin Go, Dong Cheol Song, Hyun Ah. Cho, Seung Yeol Cho, Dong Jun Kim, Mi Suk Kim, Hyeun Bum Kim, Jin Ho Cho

**Affiliations:** 1 Department of Animal Science, Chungbuk National University, Cheongju, 28644 South Korea; 2 Division of Animal and Dairy Science, Chungnam National University, Daejeon, 34134 South Korea; 3 Eugene-Bio, Suwon, 16675 South Korea; 4 Department of Animal Resources Science, Dankook University, Cheonan, 31116 South Korea

**Keywords:** Barrier integrity, *Escherichia coli*, Immunity, Phytogenic feed additive, Post-weaning diarrhea, Weaned pigs

## Abstract

**Background:**

This study was conducted to investigate the effects of each phytogenic feed additive (PFA; PFA1, bitter citrus extract; PFA2, a microencapsulated blend of thymol and carvacrol; PFA3, a mixture of bitter citrus extract, thymol, and carvacrol; PFA4, a premixture of grape seed, grape marc extract, green tea, and hops; PFA5, fenugreek seed powder) on the growth performance, nutrient digestibility, intestinal morphology, and immune response in weaned pigs infected with *Escherichia coli* (*E. coli*).

**Results:**

A total of 63 4-week-old weaned pigs were placed in individual metabolic cages and assigned to seven treatment groups. The seven treatments were as follows: 1) NC; basal diet without *E. coli* challenge, 2) PC; basal diet with *E. coli* challenge, 3) T1; PC + 0.04% PFA1, 4) T2; PC + 0.01% PFA2, 5) T3; PC + 0.10% PFA3, 6) T4; PC + 0.04% PFA4, 7) T5; PC + 0.10% PFA5. The experiments lasted in 21 d, including 7 d before and 14 d after the first *E. coli* challenge. In the *E. coli* challenge treatments, all pigs were orally inoculated by dividing a total of 10 mL of *E. coli* F18 for 3 consecutive days. The PFA-added groups significantly increased (*P* < 0.05) average daily gain and feed efficiency and decreased (*P* < 0.05) the fecal score at d 0 to 14 post-inoculation (PI). Tumor necrosis factor α was significantly lower (*P* < 0.05) in the PFA-added groups except for T1 in d 14 PI compared to the PC treatment. The T3 had a higher (*P* < 0.05) immunoglobulin G and immunoglobulin A concentration compared to the PC treatment at d 7 PI. Also, T3 showed significantly higher (*P* < 0.05) villus height:crypt depth and claudin 1 expression in ileal mucosa, and significantly down-regulated (*P* < 0.05) the expression of calprotectin compared to the PC treatment.

**Conclusions:**

Supplementation of PFA in weaned pigs challenged with *E. coli* alleviated the negative effects of *E. coli* and improved growth performance. Among them, the mixed additive of bitter citrus extract, thymol, and carvacrol showed the most effective results, improving immune response, intestinal morphology, and expression of tight junctions.

## Background

Post-weaning diarrhea (PWD) results in increased dehydration, mortality, and lowered growth performance in weaned pigs [1]. PWD is considered the main cause of economic loss in the swine industry because it destabilizes the health status of pigs, reduces production efficiency, and increases production costs as a result [2, 3]. PWD is caused by significant changes in gastrointestinal physiology, microbiology, and immunology due to weaning stress, the biggest stressor in piglets [4]. Pathogenic *Escherichia coli* (*E. coli*) is known to be a major cause of PWD [5]. *E. coli* damages the intestinal epithelium, weakening mucosal and cellular barrier functions and increasing the adhesion of pathogenic bacteria to the mucosal layer [4, 6].

Phytogenic feed additives (PFA) contain various physiologically active ingredients such as alkaloids, flavonoids, saponins, tannins phenolics, polyphenols, thymol, and allicin, and have positive activity including antibacterial, immune-modulating, antioxidant, and growth-promoting effects in animals [7, 8]. When weaned pigs challenged with *E. coli* were fed a blended plant feed additive containing naringin flavonoids, intestinal damage was prevented by reducing the main acute phase protein of pigs and better controlling the inflammatory response [9]. Also, the addition of essential oils to the diet of weaned pigs challenged with *E. coli* increased growth performance and the apparent total tract digestibility (ATTD) of nutrients and decreased the incidence of diarrhea [10].

However, no studies have compared the effects of different PFA supplements in weaned pigs infected with *E. coli* at the same time. Therefore, this study was conducted to investigate the effects of individual PFAs on growth performance, nutrient digestibility, intestinal morphology, and immune response in weaned pigs infected with *E. coli*, which is a principal causative agent of PWD, and then identify the PFA effective against PWD.

## Materials and methods

### Test phytogenic feed additives

Five types of PFA were used in this study. PFA1 is composed of bitter citrus extract (BioFlavex GC, HTBA, Beniel, Spain) and contains 25 ~ 27% naringin and 11 ~ 15% neohesperidin. PFA2 is a microencapsulated blend of thymol and carvacrol (Avipower 2, VetAgro SpA, Reggio, Emmilia, Italy), containing 7% of thymol and 7% of carvacrol. PFA3 is a mixture of PFA1, PFA2 and excipient in a ratio of 4:1:5. It contains 0.7% thymol, 0.7% carvacrol, 10 ~ 10.8% naringin and 4.4 ~ 6% neohesperidin. PFA 4 is a premixture of grape seed and grape marc extract, green tea, and hops (AntaOx Flavosyn, DR. Eckel GmbH, Niederzissen, Germany). It contains more than 10% of flavonoids. PFA5 is composed of fenugreek seed powder (Fenugreek Seed Powder, P&D Export, Jaguar, India) and contains 12% saponin. All PFAs used in this study was obtained by a commercial company (Eugene-Bio, Suwon, South Korea).

### Bacterial strains and culture

Shiga toxin-producing *E. coli* F18 was provided in stock form. The F18 *E. coli* expressed heat labile toxin (LT) and shiga toxin type 2e (stx2e). Ten microliters of thawed *E. coli* stock was inoculated into 10 mL of nutrient broth and cultured at 37 °C for 24 h, and then subcultured. Thereafter, the subcultured *E. coli* was smeared on MacConkey agar to confirm the bacterial enumeration. A final concentration of 1.2 × 10^10^ CFU/mL was used in this study.

### Animals, treatments and experimental design

A total of 63 4-week-old crossbred weanling pigs [(Landrace × Yorkshire) × Duroc] with initial body weight (BW) of 8.03 ± 0.43 kg were used in this study. All pigs were assigned to a completely randomized seven treatment groups based on the initial BW. There was one pig in a cage and nine replicate cages per treatment. Pigs were individually placed in 45 cm × 55 cm × 45 cm stainless steel metabolism cages in an environmentally controlled room. Pigs were housed in individual pens for 21 d, including 7 d before and 14 d after the first *E. coli* challenge (d 0). Dietary treatments were as follow: 1) NC (negative control; basal diet without *E. coli* challenge), 2) PC (positive control; basal diet with *E. coli* challenge), 3) T1 (PC + 0.04% PFA1), 4) T2 (PC + 0.01% PFA2), 5) T3 (PC + 0.10% PFA3), 6) T4 (PC + 0.04% PFA4), 7) T5 (PC + 0.10% PFA5). All diets were formulated to meet or exceed the NRC requirement (Table 1) [11]. All treatment groups were fed the experimental diet for 21 d, including 7 d of adaptation. The diets were mixed with water in a 1:1 ratio before feeding and were fed at 08:30 and 17:30 each day. The pigs had ad libitum access to water.

**Table 1:** Compositions of basal diets (as-fed-basis)

Items	Content
Ingredients, %	
Corn	34.43
Extruded corn	15.00
Lactose	10.00
Dehulled soybean meal, 51% CP^a^	13.50
Soy protein concentrate, 65% CP^a^	10.00
Plasma powder	6.00
Whey	5.00
Soy oil	2.20
Monocalcium phosphate	1.26
Limestone	1.40
*L*-Lysine-HCl, 78%	0.06
*DL*-Methionine, 50%	0.15
Choline chloride, 25%	0.10
Vitamin premix^b^	0.25
Trace mineral premix^c^	0.25
Salt	0.40
Total	100.00
Calculated value	
ME, Kcal/kg	3433
CP, %	20.76
Lysine, %	1.35
Methionine, %	0.39
Ca	0.82
P	0.65
Analyzed value	
ME, kcal/kg	3512
CP, %	20.92

^a^*Abbreviation*: *CP* Crude protein

^b^Provided per kg of complete diet: vitamin A, 11,025 IU; vitamin D_3_, 1103 IU; vitamin E, 44 IU; vitamin K, 4.4 mg; riboflavin, 8.3 mg; niacin, 50 mg; thiamine, 4 mg; d-pantothenic, 29 mg; choline, 166 mg; and vitamin B_12_, 33 mg

^c^Provided per kg of complete diet without Zinc: Cu (as CuSO_4_•5H_2_O), 12 mg; Mn (as MnO_2_), 8 mg; I (as KI), 0.28 mg; and Se (as Na_2_SeO_3_•5H_2_O), 0.15 mg

In the *E. coli* challenge treatments, all pigs were orally inoculated by dividing a total of 10 mL of *E. coli* F18 for 3 consecutive days from d 0 post-inoculation (PI) after 7 d of adaptation.

### Sampling and measurements

#### Growth performance and fecal score

Pigs were weighed individually at the beginning (d −7), d 0 before inoculation, and d 7, 14 PI. Feed intake (FI) was recorded daily the diet supply amount and remaining amount. Average daily gain (ADG), average daily feed intake (ADFI), and feed efficiency (G:F) were calculated for each interval from d − 7 to 0, d 0 to 7 PI, d 7 to 14 PI and d 0 to 14 PI. The fecal scores were individually recorded at 08:00 and 17:00 by the same person during the entire experimental period. The fecal score was assigned as follows: 0, Normal feces; 1, Soft feces; 2, Mild diarrhea; 3, Severe diarrhea. The fecal score of each pig was calculated as an average within the period before and after the *E. coli* challenge.

#### Nutrient digestibility

Chromium oxide (Cr_2_O_3,_ 2 g/kg) was added to the diets as an indigestible marker to measure digestibility [12]. Pigs were fed diets mixed with chromium oxide for 4 consecutive days from d 4 and d 11, fresh excreta samples were collected in that period. At the same time, 9 replications of these feed samples were collected. Fresh fecal and feed samples were stored in a freezer at − 20 °C immediately after collection. At the end of the experiment, fecal samples were dried at 70 °C for 72 h and then crushed on a 1-mm screen. The procedures utilized for the determination of dry matter (DM) and crude protein (CP) digestibility were conducted with the methods by the AOAC [13]. Chromium levels were determined via UV absorption spectrophotometry (UV-1201, Shimadzu, Kyoto, Japan) using the Williams et al. [14] method. For calculating the ATTD of the nutrients, we used the following equation: Digestibility = 1 − [(Nf × Cd)/(Nd × Cf)] × 100, where Nf = concentration of nutrient in fecal, Nd = concentration of nutrient in the diet, Cd = concentration of chromium in the diet, and Cf = concentration of chromium in the fecal.

#### Complete blood count and measurement of serum immunoglobulin, interleukin and TNF-α 

Blood samples were collected from the jugular vein of all pigs before the *E. coli* challenge (d 0), and on d 7 and 14 PI. At the time of collection, blood samples were collected into vacuum tubes containing K_3_EDTA for complete blood count analysis, and nonheparinized tubes for serum analysis, respectively. After collection, serum samples were centrifuged at 3000×*g* for 20 min at 4 °C. Thereafter, the blood sample tubes were stored in − 20 °C refrigerator until analysis. The white blood cell (WBC), basophil, neutrophil, and lymphocyte levels in the whole blood were measured using an automatic blood analyzer (ADVIA 120, Bayer, NY, USA). Immunoglobulin G (IgG) and immunoglobulin A (IgA) levels were gauged using an automatic biochemistry blood analyzer (Hitachi 747; Hitachi, Tokyo, Japan). Interleukin-6 (IL-6) and tumor necrosis factor α (TNF-α) concentrations were determined using commercially available ELISA kits (Quantikine, R&D systems, Minneapolis, MN, USA) and the absorbance was measured at 450 nm.

#### Intestinal morphology

At the end of the experiment (d 14), pigs were anesthetized with carbon dioxide gas after blood sampling and euthanized by exsanguination. After euthanization, intestinal tissues of about 10 cm from the ileum (close to the ileocecal junction) were collected and fixed in 10% neutral buffered formalin (NBF; Sigma-Aldrich, St. Louis, MO, USA) for intestinal morphology and expression analysis of intestinal tight junction proteins. After cutting the intestine sample, it was dehydrated and dealcoholized. The samples were then installed on slides, treated with paraffin, and stained with hematoxylin and eosin. The slides were examined using an Olympus IX51 inverted phase-contrast microscope. Intestinal morphological measurements included the villus height (VH), crypt depth (CD), and villus height:crypt depth ratio (VH:CD).

#### Immunohistochemical staining

The expression of calprotectin and claudin 1 (CLDN1) was studied by using immunohistochemistry (IHC). The ileal tissue fixed in 10% NBF solution was cut, embedded in a paraffin wax block and sectioned to a thickness of 5 μm. After deparaffinizing the paraffin sections and rehydrating the tissues, they were placed in running tap water for 10 min. This section was reacted in 0.03% hydrogen peroxide solution for 15 min, and then dipped in distilled water (DW) for 10 min. Then, the antigen was retrieved and dipped in DW for 10 min, followed by blocking in 4% bovine serum albumin solution for 30 min. The tissue section was then incubated with primary antibodies, anti-calprotectin (1:800, Thermo Fisher Scientific, Waltham, MA, USA) and anti-claudin1 (1:200, Novus Biologicals, Minneapolis, MN, USA). Then, it was incubated with the secondary antibodies, envision anti-mouse (Dako, Santa Clara, CA, USA) and envision anti-rabbit (Dako). The sections were washed with tris-buffered saline-tween and incubated with 3,3′-diaminobenzidine (DAB) to visualize immune complexes. The processed slide sections were slide-scanned through Axio Scan Z1 (Carl Zeiss, Jena, Germany). The completed file was analyzed using Zen Image Analysis, an Axio Scan Z1 program (Zen 3.4 blue edition). Analysis was analyzed by the contrast of DAB of IHC and positive reaction color to total tissue area, and the results were expressed as a total area, a positive area, and the percentage of positive area.

### Statistical analysis

All data were analyzed via the general linear model procedures of SAS (SAS Institute, Cary, NC, USA), using each pen as the experimental unit. Differences between treatment means were determined using Tukey’s multiple range test. A probability level of *P* < 0.05 was indicated to be statistically significant, and a level of 0.05 ≤ *P* < 0.10 was considered to have such a tendency.

## Results

### Growth performance

There was no difference between treatment groups in the initial BW of pigs (Table 2). At d 7 PI, T2 showed a significantly higher (*P* < 0.05) BW than the other treatment groups. Compared with the NC treatment, the PC treatment reduced (*P* < 0.05) ADG and G:F at d 0 to 7 PI. Also, at d 0 to 7 PI, the PFA-added treatment groups showed higher (*P* < 0.05) ADG and G:F compared to the PC treatment. At d 7 to 14 PI, the T3 had a significantly lower (*P* < 0.05) ADFI than other treatments, but showed a higher tendency (*P* = 0.073) in G:F. Compared with the PC treatment, the PFA-added treatment groups significantly increased ADG and G:F (*P* < 0.05) at d 0 to 14 PI, but there was no difference in ADFI.

**Table 2:** Effect of PFAs on growth performance in weaned pigs challenged with *E. coli*^1^

Items	NC	PC	T1	T2	T3	T4	T5	SEM	*P*-value
BW, kg									
d − 7	8.02	8.02	8.07	8.00	8.04	8.03	8.04	0.151	1.000
d 0	8. 93	8.90	9.13	9.30	8.90	9.13	9.47	0.244	0.580
d 7 PI	11.13^ab^	10.00^b^	11.13^ab^	11.27^a^	10.77^ab^	10.67^ab^	11.10^ab^	0.265	0.021
d 14 PI	13.57	12.33	13.43	13.70	13.57	13.20	13.53	0.328	0.077
d − 7 to 0									
ADG, g	130.00	125.71	152.38	185.24	122.86	157.62	204.28	28.038	0.298
ADFI, g	293.81^ab^	257.62^b^	288.57^ab^	308.57^ab^	298.10^ab^	318.09^a^	289.76^ab^	12.821	0.055
G:F, g/g	0.43	0.49	0.53	0.60	0.39	0.42	0.67	0.082	0.185
d 0 to 7 PI									
ADG, g	314.28^a^	157.14^c^	285.72^ab^	280.95^ab^	266.67^ab^	219.05^bc^	233.33^abc^	19.048	0.001
ADFI, g	400.95	391.43	400.95	397.62	398.81	363.81	394.76	10.548	0.178
G:F, g/g	0.79^a^	0.40^c^	0.71^ab^	0.71^ab^	0.67^ab^	0.59^b^	0.58^b^	0.042	0.001
d 7 to 14 PI									
ADG, g	347.62	333.34	328.57	347.62	400.00	361.90	347.62	17.612	0.115
ADFI, g	570.48^ab^	568.10^ab^	570.95^a^	570.00^ab^	564.76^b^	570.95^a^	571.43^a^	1.365	0.013
G:F, g/g	0.61	0.59	0.58	0.61	0.71	0.63	0.61	0.030	0.073
d 0 to 14 PI									
ADG, g	330.95^a^	245.24^b^	307.15^a^	314.29^a^	333.33^a^	290.48^ab^	290.47^ab^	13.626	0.001
ADFI, g	485.71	479.76	485.95	483.81	481.78	467.38	483.10	5.459	0.239
G:F, g/g	0.68^a^	0.51^b^	0.63^a^	0.65^a^	0.69^a^	0.62^ab^	0.60^ab^	0.025	0.001

^1^*Abbreviations*: *NC* Basal diet without *E. coli* challenge (negative control), *PC* Basal diet with *E. coli* challenge (positive control), *T1* PC + 0.04% PFA1, *T2* PC + 0.01% PFA2, *T3* PC + 0.10% PFA3, *T4* PC + 0.04% PFA4, *T5* PC + 0.10% PFA5, *BW* Body weight, *ADG* Average daily gain, *ADFI* Average daily feed intake, *G:F* Feed efficiency, *PI* Post-inoculation, *SEM* Standard error of mean

^a-c^Means with different letters are significantly differ (*P* < 0.05)

### Fecal score

Before *E. coli* inoculation, there was no difference in the fecal score at d −  7 to 0 (Table 3). The fecal score from d 0 to 7 PI was significantly higher (*P* < 0.05) in the PC treatment than in other treatment groups. Also, in d 0 to 14 PI, the PC treatment showed significantly higher (*P* < 0.05) fecal scores than other treatment groups, and the PFA-added treatment groups showed similar or lower (*P* < 0.05) fecal scores to the NC treatment.

**Table 3:** Effect of PFAs on fecal score in weaned pigs challenged with *E. coli*^1^

Items	NC	PC	T1	T2	T3	T4	T5	SEM	*P*-value
d − 7 to 0									
Fecal score^2^	1.05	1.00	0.95	0.98	0.98	0.95	0.98	0.082	0.985
d 0 to 7 PI									
Fecal score	0.98^ab^	1.26^a^	1.07^ab^	0.78^b^	0.95^ab^	0.81^b^	0.84^b^	0.078	0.001
d 7 to 14 PI									
Fecal score	0.25	0.35	0.29	0.23	0.25	0.27	0.25	0.045	0.561
d 0 to 14 PI									
Fecal score	0.61^ab^	0.81^a^	0.68^ab^	0.51^b^	0.60^ab^	0.54^b^	0.54^b^	0.054	0.005

^1^*Abbreviations*: *NC* Basal diet without *E. coli* challenge (negative control), *PC* Basal diet with *E. coli* challenge (positive control), *T1* PC + 0.04% PFA1, *T2* PC + 0.01% PFA2, *T3* PC + 0.10% PFA3, *T4* PC + 0.04% PFA4, *T5* PC + 0.10% PFA5, *PI* Post-inoculation, *SEM* Standard error of mean

^2^Fecal score was determined as follow: 0, Normal feces; 1, Soft feces; 2, Mild diarrhea; 3, Severe diarrhea

^a,b^Means with different letters are significantly differ (*P* < 0.05)

### Nutrient digestibility

At d 7 PI, there were no significant differences in DM and CP digestibility between treatment groups (Table 4). The DM digestibility was significantly higher (*P* < 0.05) in the PFA-added treatment groups at d 14 PI compared to the PC treatment. No difference was observed in CP digestibility at d 14 PI among treatment groups.

**Table 4:** Effect of PFAs on nutrient digestibility in weaned pigs challenged with *E. coli*^1^

Items	NC	PC	T1	T2	T3	T4	T5	SEM	*P*-value
d 7 PI									
DM, %	82.48	81.97	81.93	81.21	82.40	81.44	81.74	0.499	0.521
CP, %	73.86	72.61	73.05	74.03	72.99	73.23	73.40	0.551	0.558
d 14 PI									
DM, %	79.21^ab^	75.62^c^	79.28^ab^	78.80^b^	81.78^a^	78.38^bc^	78.83^b^	0.678	0.001
CP, %	77.01	75.60	76.05	76.88	76.46	76.23	76.28	0.460	0.375

^1^*Abbreviations*: *NC* Basal diet without *E. coli* challenge (negative control), *PC* Basal diet with *E. coli* challenge (positive control), *T1* PC + 0.04% PFA1, *T2* PC + 0.01% PFA2, *T3* PC + 0.10% PFA3, *T4* PC + 0.04% PFA4, *T5* PC + 0.10% PFA5, *PI* Post-inoculation, *DM* Dry matter, *CP* Crude protein, *SEM* Standard error of mean

^a,b,c^Means with different letters are significantly differ (*P* < 0.05)

### Blood profile

WBC, basophil, neutrophil, and lymphocyte levels did not differ significantly between treatment groups on d 0 before *E. coli* inoculation (Table 5). The number of WBCs in whole blood was significantly lower (*P* < 0.05) in T2 and NC treatment at d 7 PI compared to other treatment groups. Also, T2 had a significantly lower percentage (*P* < 0.05) of neutrophils and a significantly higher percentage (*P* < 0.05) of lymphocytes at d 7 PI. The neutrophils and lymphocytes of the PFA-added treatment groups except for T1 were recovered to the NC treatment level at d 14 PI, and among them, T2 showed significantly similar values (*P* < 0.05) to the NC treatment.

**Table 5:** Effect of PFAs on blood profile in weaned pigs challenged with *E. coli*^1^

Items	NC	PC	T1	T2	T3	T4	T5	SEM	*P*-value
d 0									
WBC, 10^3^/μL	18.68	19.69	19.93	18.65	19.85	20.22	18.59	1.123	0.882
Basophil, %	0.50	0.47	0.47	0.50	0.53	0.57	0.53	0.039	0.493
Neutrophil, %	54.23	56.13	53.57	54.43	53.53	57.30	54.67	1.515	0.542
Lymphocyte, %	38.47	36.13	38.33	39.53	37.47	37.67	39.40	1.359	0.608
d 7 PI									
WBC, 10^3^/μL	19.21^b^	24.44^ab^	22.76^ab^	21.85^b^	24.10^ab^	28.85^a^	23.57^ab^	1.532	0.004
Basophil, %	0.77	0.60	0.67	0.60	0.73	0.73	0.73	0.052	0.128
Neutrophil, %	43.13^b^	57.30a^a^	48.17^b^	42.33^b^	50.43^ab^	47.97^b^	49.30^ab^	2.039	0.001
Lymphocyte, %	46.07^ab^	31.97^c^	41.93^ab^	48.47^a^	42.13^ab^	39.30^bc^	42.20^ab^	2.045	0.001
d 14 PI									
WBC, 10^3^/μL	13.91^c^	18.11^bc^	21.51^ab^	18.86^abc^	21.75^ab^	23.85^a^	22.37^ab^	1.302	0.001
Basophil, %	0.60^ab^	0.70^ab^	0.47^b^	0.57^ab^	0.50^b^	0.47^b^	0.80^a^	0.082	0.044
Neutrophil, %	34.50^bc^	42.37^ab^	49.60^a^	30.87^c^	39.43^bc^	42.87^ab^	36.67^bc^	2.016	0.001
Lymphocyte, %	57.23^a^	46.20^c^	45.87^c^	56.20^ab^	51.57^abc^	47.50^bc^	47.00^c^	2.071	0.001

^1^*Abbreviations*: *NC* Basal diet without *E. coli* challenge (negative control), *PC* Basal diet with *E. coli* challenge (positive control), *T1* PC + 0.04% PFA1, *T2* PC + 0.01% PFA2, *T3* PC + 0.10% PFA3, *T4* PC + 0.04% PFA4, *T5* PC + 0.10% PFA5, *PI* Post-inoculation, *WBC* White blood cell, *SEM* Standard error of mean

^a-c^Means with different letters are significantly differ (*P* < 0.05)

### Serum immunoglobulin

In serum IgG, IgA, TNF-α, and IL-6, there was no significant difference between treatment groups at d 0 before *E. coli* inoculation (Table 6). At d 7 PI, T3 had a higher IgG and IgA concentration (*P* < 0.05) compared to PC treatment. TNF-α was significantly lower (*P* < 0.05) in the PFA-added treatment groups than in the PC treatment at d 7 PI, among which T3 and T5 were the lowest (*P* < 0.05). At d 7 PI, T3 showed the lowest IL-6 (*P* < 0.05) compared to other treatments. At d 14 PI, the IgG concentration showed a tendency for T3 to be higher (*P* = 0.077) than the PC treatment. TNF-α was significantly lower (*P* < 0.05) in the PFA-added treatment groups except for T1 in d 14 PI compared to the PC treatment.

**Table 6:** Effect of PFAs on immunoglobulins and cytokines in weaned pigs challenged with *E. coli*^1^

Items	NC	PC	T1	T2	T3	T4	T5	SEM	*P*-value
d 0									
IgG, mg/dL	219.67	215.00	220.33	215.33	216.67	219.33	218.67	9.251	0.999
IgA, mg/dL	1.67	1.67	1.33	1.33	1.67	1.67	1.33	0.167	0.350
TNF-α, pg/mL	43.80	43.08	44.20	40.11	41.53	42.39	41.09	1.874	0.704
IL-6, pg/mL	50.93	53.30	52.43	55.30	51.20	52.37	50.33	5.217	0.996
d 7 PI									
IgG, mg/dL	177.67^a^	127.00^c^	143.33^bc^	153.67^abc^	157.67^ab^	149.00^bc^	147.67^bc^	8.809	0.012
IgA, mg/dL	1.33^a^	1.00^b^	1.00^b^	1.00^b^	1.33^a^	1.33^a^	1.00^b^	0.109	0.024
TNF-α, pg/mL	37.14^b^	51.55^a^	30.71^bc^	28.82^bc^	26.86^c^	30.12^bc^	24.47^c^	2.098	0.001
IL-6, pg/mL	49.58^ab^	57.50^a^	40.93^ab^	46.47^ab^	32.43^b^	50.90^ab^	49.33^ab^	4.360	0.007
d 14 PI									
IgG, mg/dL	178.33	143.67	148.67	171.00	176.67	160.00	153.00	9.775	0.077
IgA, mg/dL	1.33	1.33	1.00	1.33	1.33	1.33	1.00	0.141	0.258
TNF-α, pg/mL	38.46^ab^	47.25^a^	46.62^a^	30.37^ab^	23.67^b^	22.65^b^	23.33^b^	4.000	0.001
IL-6, pg/mL	44.20	45.27	34.40	29.87	38.90	37.43	44.27	4.133	0.087

^1^*Abbreviations*: *NC* Basal diet without *E. coli* challenge (negative control), *PC* Basal diet with *E. coli* challenge (positive control), *T1* PC + 0.04% PFA1, *T2* PC + 0.01% PFA2, *T3* PC + 0.10% PFA3, *T4* PC + 0.04% PFA4, *T5* PC + 0.10% PFA5, *PI* Post-inoculation, *IgG* Immunoglobulin G, *IgA* Immunoglobulin A, *TNF-α* Tumor necrosis factor α, *IL-6* Interleukin-6, *SEM* Standard error of mean

^a -c^Means with different letters are significantly differ (*P* < 0.05)

### Intestinal morphology

**Fig. 1:**
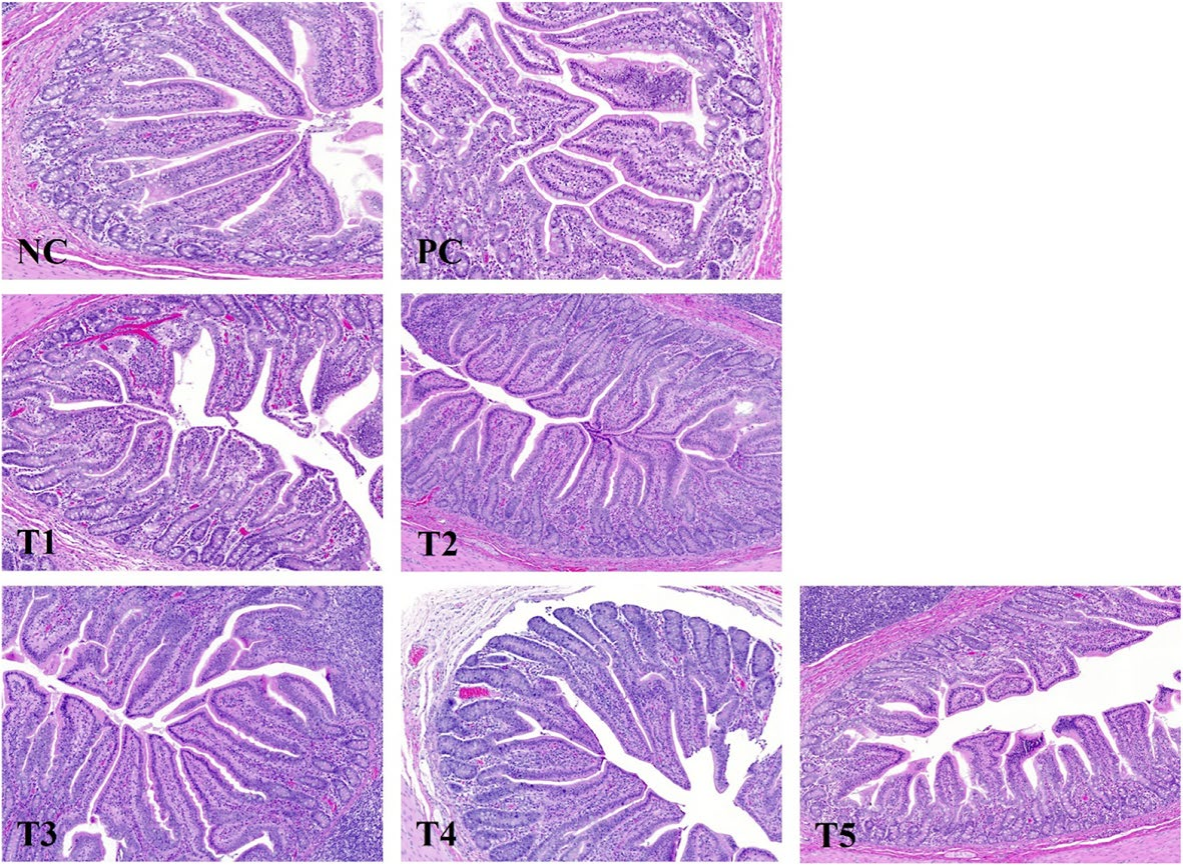
Effect of PFAs on intestinal microscopic morphology in weaned pigs challenged with *E. coli*. NC, basal diet without *E. coli* challenge (negative control); PC, basal diet with *E. coli* challenge (positive control); T1, PC + 0.04% PFA1; T2, PC + 0.01% PFA2; T3, PC + 0.10% PFA3; T4, PC + 0.04% PFA4; T5, PC + 0.10% PFA5. Scale bar is 100 μm

VH in T3 and NC treatment was significantly higher (*P* < 0.05) than in PC treatment (Table 7; Fig. 1). In CD, the PFA-added treatment groups showed a tendency to be numerically lower (*P* = 0.067) than those of the PC treatment. VH:CD showed significantly higher values (*P* < 0.05) in T3 than PC treatment.

**Table 7:** Effect of PFAs on intestinal morphology in weaned pigs challenged with *E. coli*^1^

Items	NC	PC	T1	T2	T3	T4	T5	SEM	*P*-value
VH, μm	376.88^a^	324.45^b^	334.45^ab^	351.12^ab^	371.27^a^	360.95^ab^	334.93^ab^	10.453	0.004
CD, μm	141.55	175.57	165.69	157.20	148.86	150.40	174.68	9.115	0.067
VH:CD	2.69^a^	1.89^c^	2.07^abc^	2.30^abc^	2.59^ab^	2.44^abc^	1.99^bc^	0.147	0.001

^1^*Abbreviations*: *NC* Basal diet without *E. coli* challenge (negative control), *PC* Basal diet with *E. coli* challenge (positive control), *T1* PC + 0.04% PFA1, *T2* PC + 0.01% PFA2, *T3* PC + 0.10% PFA3, *T4* PC + 0.04% PFA4, *T5* PC + 0.10% PFA5, *PI* Post-inoculation, *VH* Villus height, *CD* Crypt depth, *VH:CD* Villus height: Crypt depth, *SEM* Standard error of mean

^a-c^Means with different letters are significantly differ (*P* < 0.05)

### Tight junction

T3 and NC treatment significantly down-regulated (*P* < 0.05) the expression of calprotectin in the ileum mucosa compared to other treatments (Table 8). The expression of CLDN1 in the ileal mucosa was significantly higher (*P* < 0.05) in the PFA-added treatment groups than in the PC treatment, and the T3 showed the highest (*P* < 0.05) among the PFA-added treatment groups.

**Table 8:** Effect of PFAs on expression of tight junction proteins in weaned pigs challenged with *E. coli*^1^

Items	NC	PC	T1	T2	T3	T4	T5	SEM	*P*-value
Calprotectin, %	0.033^c^	0.100^a^	0.084^ab^	0.066^abc^	0.042^c^	0.057^bc^	0.062^abc^	0.004	0.001
CLDN1, %	18.24^a^	8.48^d^	13.86^c^	14.42^c^	17.72^ab^	15.63^bc^	15.78^abc^	0.440	0.001

^1^*Abbreviations*: *NC* Basal diet without *E. coli* challenge (negative control), *PC* Basal diet with *E. coli* challenge (positive control), *T1* PC + 0.04% PFA1, *T2* PC + 0.01% PFA2, *T3* PC + 0.10% PFA3, *T4* PC + 0.04% PFA4, *T5* PC + 0.10% PFA5, *PI* Post-inoculation, *CLDN1* Claudin 1, *SEM* Standard error of mean

^a-d^Means with different letters are significantly differ (*P* < 0.05)

## Discussion

*E. coli*-induced PWD is a common intestinal disease in weaned pigs, causing economic loss by reducing growth performance and increasing mortality [15]. This disease usually occurs in the early weaning of pigs at 3 to 4 weeks of age, and the symptoms appear between 3 and 10 d after weaning [16, 17]. In the results of this study, after the *E. coli* challenge, crypt depth and the expression of calprotectin were increased, and the villus height was decreased compared to the NC treatment. Also, 61% of pigs in this study had diarrhea for 7 d after the *E. coli* challenge. These observations are consistent with those seen in weaned pigs infected with *E. coli* in previous studies [18–20]. Therefore, these obvious clinical signs and symptoms indicated that the pigs were successfully infected with *E. coli*.

Our study was performed to assess the effects of several PFAs added to the diets of *E. coli*-infected weaned pigs and to determine which PFAs were effective for PWD. The results of this study indicated that all PFAs had a positive effect on the growth performance of *E. coli*-infected weaned pigs. The findings suggest that PFAs may help weaned pigs cope with stress during the post-weaning phase when impaired pig growth performance most commonly occurs. Naringin and neohesperidin are the major antioxidants in citrus fruits, with naringin, in particular, reported to have anti-inflammatory and antioxidant properties [21, 22]. Through this action, ADG and the feed efficiency of weaned pigs were improved when naringin was added to the diet of weaned pigs in our study, as well as in a previous study [23]. According to Windisch et al. [24], essential oils, including thymol and carvacrol, might enhance the activity of digestive enzymes and hence, increase nutritional absorption, resulting in a higher feed efficiency. This is consistent with the results of the current study. Also, the addition of essential oils reduced the incidence of diarrhea by 50% [25]. Previous studies reported that both thymol and carvacrol had antibacterial and anti-inflammatory effects [26, 27]. According to Ouwehand et al. [28], beneficial microorganisms like *Lactobacilli* and *Bifidobacteria* were less susceptible to the antibacterial activity of essential oils than potentially harmful bacteria like *E. coli* and *Salmonella*. For this reason, in this study, it seems that a microencapsulated blend of thymol and carvacrol reduced the fecal scores by causing the positive modulation of intestinal microflora. Although no previous studies on bitter citrus extract, thymol, and carvacrol mixed additives have been conducted, the results in the present study suggest that the positive effects such as antibacterial, anti-inflammatory, and antioxidant properties in the components of each additive may have alleviated the adverse effects of *E. coli* challenge. In particular, this mechanism can explain the improvement of the T3 group’s growth performance and fecal scores.

In the case of nutrient digestibility, there was no significant difference between the treatment groups 7 d after the *E. coli* challenge. However, after 14 d, DM digestibility was higher in the PFA-added treatment groups than in the PC treatment. The enhanced digestive capacity of the small intestine could be attributed to PFA, which stabilizes microbial eubiosis in the gut [29]. Previous studies showed that polyphenols helped digestion by reducing inflammation and increasing the synthesis of digestive enzymes [30]. PWD-induced weaned pigs fed fenugreek extract showed higher DM digestibility than pigs fed the basal diet after an *E. coli* challenge [29]. In the present study, the T3 group fed a mixture of bitter citrus extract, thymol and carvacrol showed the highest DM digestibility on 14 d after the *E. coli* challenge. This is considered to be a synergistic effect of the addition of each PFA. There was no significant difference in CP digestibility between the treatment groups at both d 7 PI and d 14 PI. However, when CP digestibility was viewed only as a numerical value, and not a statistical difference, supplementation with thymol and carvacrol showed a digestibility similar to or higher than that of the NC treatment that was not subjected to an *E. coli* challenge. These results indicate that the addition of essential oils to *E. coli*-infected pigs could improve nutrient absorption by stimulating saliva and bile secretion and increasing enzymatic activity [8, 31].

An increase in the total number of WBCs indicates the presence of systemic inflammation [32]. Lymphocytes in the whole blood provide specific cellular and humoral immune responses, whereas neutrophils serve as a first-line defense against bacterial infections [17]. Their ratio is commonly utilized as a biomarker to diagnose systemic inflammation severity [32]. According to Liu et al. [33], an *E. coli* challenge could induce systemic inflammation in weaned pigs by increasing the total number of WBCs and neutrophils. The present study also confirmed that the *E. coli* challenge increased WBCs and neutrophils and decreased lymphocytes at both d 7 PI and d 14 PI. Among them, supplementation with thymol and carvacrol showed the same or significantly improved WBCs, neutrophil, and lymphocyte counts at d 7 PI, and neutrophil and lymphocyte counts at d 14 PI compared to the NC treatment not infected with *E. coli*. This suggests that the addition of thymol and carvacrol to weaned pigs infected with *E. coli* could attenuate systemic inflammation caused by an *E. coli* infection. Similarly, in pigs challenged with lipopolysaccharide (LPS) from *E. coli*, the addition of essential oils showed improved WBCs and neutrophil results compared to pigs fed the basal diet after the LPS challenge due to the anti-inflammatory response to essential oils [34]. 

Serum IgA and IgG are important immunoglobulins in humoral immunity [35]. In particular, IgG has been found in high amounts in the serum in response to external infection, with a lengthy half-life [36]. Due to weaning stress and the immaturity of the piglet immune system, a reduction in serum IgG concentrations normally occurs during the weaning phase [37]. The study results showed that the *E. coli* challenge significantly decreased serum IgG and IgA concentrations at d 7 PI compared to the NC treatment, and showed a decreased tendency at d 14 PI. Furthermore, the feeding of a mixture of bitter citrus extract, thymol and carvacrol to pigs infected with *E. coli* showed significantly higher IgG and IgA concentrations at both d 7 PI and d 14 PI compared to PC treatment. Ahmed et al. [38] reported that the feeding of essential oils in the *E. coli* challenge condition increased the IgG levels of weaned pigs, consistent with the results of the present study. Thymol contained in essential oils can increase goblet cells in the animal ileum and inhibit the growth of pathogenic bacteria [39, 40]. Also, the addition of essential oils could improve immunity by regulating the humoral immune system of weaned pigs [41]. The mechanism by which PFA increases IgG levels requires further study, but it has been suggested that active phytogenic (flavonoids and polyphenols) molecules may act as additional ligands of Fc receptors to bind IgG and stimulate immune responses [42]. Cytokines are also involved in the maintenance of immune homeostasis [35]. The pro-inflammatory cytokines IL-6 and TNF -α are frequently used as potential markers in weaning pigs to diagnose pathogenic infections [43]. TNF- α and IL-6 impair animal performance by impairing immunity and nutrient metabolism [44]. This could emphasize the crucial role of cytokines in the regulation of immune and inflammatory responses. In the present study, the *E. coli* challenge significantly increased TNF-α at both d 7 PI and d 14 PI. Also, the *E. coli* challenge increased IL-6 at d 7 PI and showed a high trend at d 14 PI. However, both TNF-α and IL-6 were significantly lower in the T3 group fed a mixture of bitter citrus ext ract, thymol and carvacrol than in the PC treatment, suggesting that this PFA positively modulated the immune and inflammatory responses. TNF-α levels in the blood have been linked to gut inflammatory disease, and a previous study found that a combination of carvacrol and thymol lowered *TNF-α* mRNA expression in the intestine of weaned pigs, leading to improved gut health and growth performance [45]. Therefore, the present results suggest tha t the addition of a mixture of bitter citrus extract, thymol, and carvacrol could suppress the inflammatory response caused by *E. coli* in weaned pigs and have a beneficial effect on health status.

Intestinal morphology is commonly assessed by measuring VH, CD, and the VH:CD ratio [46]. Intestinal morphology can reveal some information on gut health. A shortening of the villus and deeper crypts may decrease the surface area for nutrient absorption. Therefore, the VH:CD ratio value is utilized as a useful measure of nutrient digestion and absorption [47]. The *E. coli* inoculum used in the present study expresses LT and Stx2e, which could induce intestinal morphological lesions such as villus atrophy and leaky gut in weaned pigs [33, 48]. These lesions impair nutrient absorption and are a major cause of reduced growth performance [49, 50]. In this study, the *E. coli* challenge also reduced the VH:CD ratio, indicating the deterioration of intestinal health. However, supplementation with a mixture of bitter citrus extract, thymol, and carvacrol improved VH, CD, and the VH:CD ratio in pigs compared to those in the PC treatment. Essential oils reduce the number of pathogenic bacteria in the gut through their antibacterial action [28]. It has been reported that essential oils can improve epithelial cell proliferation to build intestinal villus and improve intestinal morphology [51]. In addition, the antioxidant effect could decrease the intestinal damage caused by oxidative stress and improve the intestinal morphology of weaned pigs [52]. Therefore, the improvement in intestinal morphology in the T3 group was thought to be due to the complex antibacterial and antioxidant actions of thymol, carvacrol, and bitter citrus extract.

The intestinal epithelia, especially the internal tight junctions such as occludin and CLDN1 between enterocytes, are critical in maintaining intestinal permeability [53]. Enteric infections and endotoxin translocation can increase the permeability of the intestinal epithelium by altering tight junctions [54]. A disruption in intestinal barrier function might also be caused by a disruption in the expression of tight junction proteins [1, 55]. As a result of the diminished intestinal integrity, bacterial translocation is enhanced, and cellular pro-inflammatory responses stimulated by foreign invading bacteria might be increased [15, 56, 57]. Calprotectin mainly plays a role in preventing the binding of bacteria to mucosal epithelial cells through zinc competition and is used as an indicator to quantify the extent of intestinal inflammation in infectious and inflammatory diseases [19, 58]. In the current study, supplementation with bitter citrus extract, thymol, and carvacrol down-regulated the expression of calprotectin and up-regulated the expression of CLDN1 in the ileal mucosa on d 14 PI. A previous study reported that feeding guava leaf extract containing naringin to weaned pigs challenged with *E. coli* improved intestinal permeability and mucosal damage caused by *E. coli* and improved tight junction integrity [59]. The down-regulation of calprotectin could be seen as an indicator of attenuated intestinal inflammation caused by PFA. Therefore, these results indicated that supplementation with bitter citrus extract, thymol, and carvacrol helped to maintain normal intestinal integrity and immune functions, enhancing disease resistance and the performance of *E. coli*-challenged pigs.

## Conclusion

In weaned pigs infected with *E. coli*, the addition of PFA alleviated the negative effects of *E. coli* and improved growth performance. When supplemented with thymol and carvacrol, the antibacterial and anti-inflammatory effects of thymol and carvacrol have been shown to prevent diarrhea. When a mixture of bitter citrus extract, thymol, and carvacrol was fed, immunity, intestinal morphology, and tight junction expression were all improved. Therefore, when a mixture of bitter citrus extract, thymol, and carvacrol is added, each PFA effect seems to be added and synergistic, and it is considered that this mixed additive is the most effective PFA among the five additives used in the study. However, it seems that additional studies are needed on the proper amount of each additive and the basic mechanism of PFA against PWD.

## Data Availability

The datasets from the current study are available from the corresponding author upon reasonable request.

## Abbreviations

ADFI: Average daily feed intake
ADG: Average daily gain
ATTD: Apparent total tract digestibility
BW: Body weight
CD: Crypt depth
CLDN1: Claudin 1
CP: Crude protein
DAB: 3,3′-diaminobenzidine
DM: Dry matter
DW: Distilled water
*E. coli*: *Escherichia coli*
FI: Feed intake
G:F: Feed efficiency
IgA: Immunoglobulin A
IgG: Immunoglobulin G
IHC: Immunohistochemistry
IL-6: Interleukin-6
LPS: Lipopolysaccharide
LT: Heat labile toxin
NBF: Neutral buffered formalin
NC: Negative control
PC: Positive control
PFA: Phytogenic feed additive
PI: Post-inoculation
PWD: Post-weaning diarrhea
Stx2e: Shiga toxin type 2e
TNF-α: Tumor necrosis factor α
VH: Villus height
VH:CD: Villus height:crypt depth
WBC: White blood cell
